# Editorial: Advances in pharmacological treatments for urogenital disorders

**DOI:** 10.3389/fruro.2026.1776078

**Published:** 2026-01-29

**Authors:** Xiaolong Wang, Peng Wang, Qingfeng Yu

**Affiliations:** 1 Department of Pathology, Lewis Katz School of Medicine, Temple University, Philadelphia, PA, United States; 2 Coriell Institute for Medical Research, Camden, NJ, United States; 3 Department of Urology, The First Affiliated Hospital of Guangzhou Medical University, Guangzhou, China

**Keywords:** artificial intelligence - AI, bladder cancer (BCa), BPH (benign prostatic hyperplasia), combination therapy, pharmacological treatment

Pharmacological treatments have become increasingly central to managing urogenital disorders, including benign prostatic hyperplasia, bladder inflammation, and bladder cancer, progressively reducing reliance on invasive surgery. Contemporary strategies integrate targeted pharmacological agents with natural supplements, artificial intelligence (AI)-driven drug selection, and regenerative medicine approaches including plant-derived bioactive compounds. This combination approach transcends simple co-administration of agents, instead of representing intelligent integration of multiple modalities for synergistic therapeutic benefits. Furthermore, urology’s historic tradition of technological innovation, superior patient treatment adherence, and the specialty’s progressive mindset collectively position it as an ideal platform for translating innovative pharmacological and regenerative medicine strategies into clinical practice (1).

Zhou et al. have eloquently articulated the paradigm shift in BPH management, demonstrating that treatment is transitioning from single-modality, traditional surgical approaches such as transurethral resection of the prostate (TURP) toward multi-modal, patient-centered models. Emerging minimally invasive surgical therapies (MISTs), particularly those designed to preserve sexual function and accommodate variable prostate volumes, are gaining prominence. Concurrently, future therapeutic approaches will increasingly incorporate personalized treatment strategies that leverage AI and regenerative medicine, enabling clinicians to select optimal interventions based on individual patient characteristics including prostate size, symptom severity, and comorbidity profiles. AI technologies are poised to become transformative agents in BPH management, fundamentally reshaping treatment selection and prognostication.

Cellular and immunological targeted drug therapies provide viable alternatives for patients unable to tolerate conventional treatments. Lanman et al. have identified TREM2-high and MARCO-high macrophages as key drivers of lower urinary tract symptoms in patients with enlarged prostates, revealing that lipid-rich macrophages exacerbate disease progression. This finding reveals how lipid metabolism dysregulation drives chronic BPH-associated inflammation and hyperplasia, establishing a molecular framework for TREM2-targeted therapeutics. For patients unsuitable for surgery or unresponsive to monotherapy, macrophage-targeted combination approaches offer promising options for the patients, particularly in metabolic syndrome-related BPH resistant to current treatments like α-blockers and 5α-reductase inhibitors (2).

While cystoscopy remains the gold standard diagnostic tool in bladder disease management, combination anesthetic pharmacological preparation has substantially improved procedural efficacy and patient tolerance. Bai et al. have documented that combined anesthesia—integrating tetracaine hydrochloride topical surface anesthesia with intravenous anesthesia—provides substantial advantages for painless cystoscopy. This pharmacological combination effectively reduces propofol dosage requirements, shortens both cystoscopic and total operative times by approximately 50%, and simultaneously improves postoperative oxygenation (SpO_2_) while maintaining excellent safety profiles. These outcomes translate into enhanced patient trust and satisfaction, fundamental elements that strengthen the therapeutic relationship and procedural acceptance.

In bladder cancer management, while surgical approaches represent established standards of care, pharmacological treatments remain critically important and can be strategically combined with plant-derived therapeutics. Guo et al. have recently demonstrated that dihydromyricetin (DHM), a natural plant-derived compound, exhibits potent anti-tumor activity against muscle-invasive bladder cancer (MIBC) through multiple mechanisms: inhibiting tumor proliferation and metastasis by inducing cell cycle arrest, activating caspase-dependent apoptotic pathways, and reversing the epithelial-mesenchymal transition (EMT) process. Although certain dosages demonstrate potential pulmonary toxicity, the overall favorable efficacy-to-toxicity profile positions DHM as a strong candidate for combination chemotherapy approaches.

**Figure 1:**
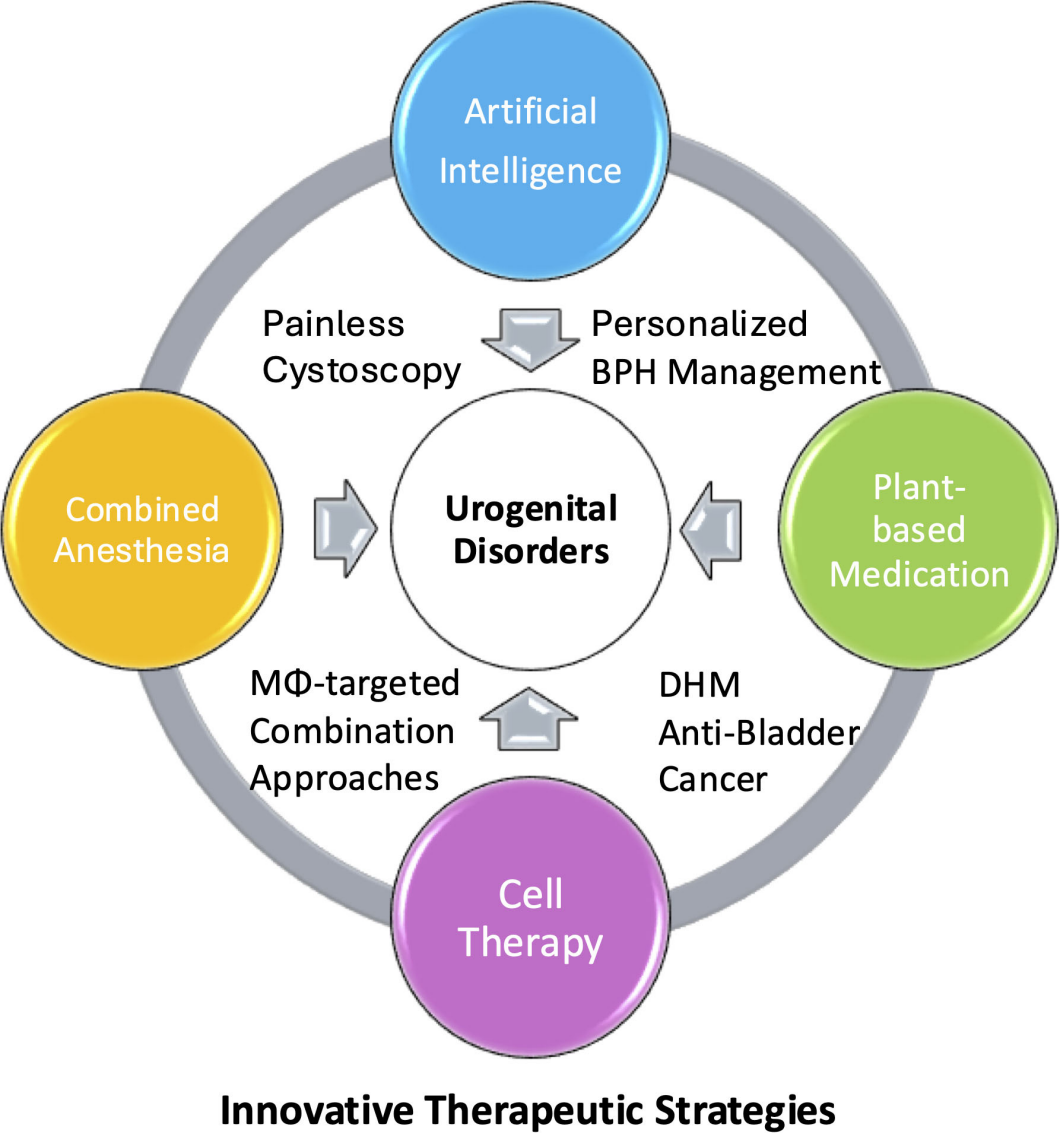
Advances in combination therapy for urogenital disorders. The integrated framework illustrates four cutting-edge therapeutic approaches that are advancing the treatment of urogenital disorders. First, artificial intelligence has emerged as a powerful tool for personalizing BPH treatment protocols while also enhancing the feasibility of painless cystoscopy procedures. Second, plant-derived therapeutics represent a promising medication, with compounds such as dihydromyricetin (DHM) showing potential in bladder cancer treatment. Third, cellular therapy approaches are being explored through macrophage-targeted (MΦ-targeted) combination strategies that address a group of urogenital pathologies. Finally, innovation in combined anesthesia protocols have proven instrumental in achieving painless cystoscopy and improving overall patient comfort during diagnostic and therapeutic interventions. These four domains are inherently interconnected, demonstrating how digital technology, novel pharmacology, cellular bioengineering, and procedural innovations work together to create comprehensive solutions for contemporary urological and genital health challenges.

The overarching principle unifying these diverse advances is the centrality of combination therapy (**Figure 1**). Whether through AI-enhanced clinical decision-making, combined anesthetic techniques, or the integration of plant-derived compounds with conventional pharmacotherapy, the evidence consistently supports multimodal approaches over isolated interventions across all urogenital pathologies. This combination philosophy must guide our therapeutic strategy at every level—from diagnosis through clinical practice (3).

Urogenital disorders inherently benefit from combination therapy due to their multifactorial pathophysiology and complex patient populations. These conditions—erectile dysfunction, lower urinary tract symptoms, infertility, and chronic pelvic pain—rarely stem from a single cause; they involve vascular, neurogenic, hormonal, psychological, and inflammatory components acting in concert. Furthermore, urogenital patients are predominantly aging adults with significant comorbidities (cardiovascular disease, diabetes, hypertension and other metabolic syndrome) that directly influence disease pathogenesis and therapeutic response (4). Combination therapy’s simultaneous targeting of multiple disease pathways proves superior to monotherapy, particularly in aging patients with complex urogenital disorders where single interventions prove inadequate. This multimodal approach addresses the intrinsic complexity of urogenital pathology and yields better clinical outcomes. This foundation supports a fundamental paradigm shift in urological practice: from asking “Is surgery indicated?” to “Which combination of interventions—pharmacological, surgical, behavioral, or technological—will optimally serve this individual patient?”.
